# A novel peptide ADAM8 inhibitor attenuates bronchial hyperresponsiveness and Th2 cytokine mediated inflammation of murine asthmatic models

**DOI:** 10.1038/srep30451

**Published:** 2016-07-26

**Authors:** Jun Chen, Linhong Deng, Daniela Dreymüller, Xuemei Jiang, Jiaoyue Long, Yiyuan Duan, Yue Wang, Mingzhi Luo, Feng Lin, Lizhen Mao, Bernd Müller, Garrit Koller, Jörg W. Bartsch

**Affiliations:** 1 Changzhou Key Laboratory of Respiratory Medical Engineering, Institute of Biomedical Engineering and Health Sciences, Changzhou University, Changzhou, Jiangsu, China; 2 Key Lab of Biorheological Science and Technology, Ministry of Education, Bioengineering College, Chongqing University, Chongqing, China; 3 Institute of Pharmacology and Toxicology, RWTH Aachen University, Wendlingweg 2, 52074 Aachen, Germany; 4 Jiangsu Asialand Bio-med Technology Co. Ltd., Changzhou, Jiangsu, China; 5 Laboratory of Respiratory Cell Biology, Division of Pneumology, Philipps-University Marburg, Marburg, Germany; 6 KCLDI Biomaterials, Biomimetics and Biophotonics Group. King’s College London, London SE1 9RT, United Kingdom; 7 Department of Neurosurgery, Philipps-University Marburg, Baldinger Str., 35033 Marburg, Germany

## Abstract

A disintegrin and metalloproteinase 8 (ADAM8) has been identified as a signature gene associated with moderate and severe asthma. Studies in mice have demonstrated that the severity of asthma can be reduced by either transgenic knock-out or by antibodies blocking ADAM8 function, highlighting ADAM8 as potential drug target for asthma therapy. Here, we examined the therapeutic effect of an ADAM8 inhibitor peptide (BK-1361) that specifically blocks cellular ADAM8 activity in ovalbumin-sensitized and challenged Balb/c mice. We found that BK-1361 (25 μg/g body weight) attenuated airway responsiveness to methacholine stimulation by up to 42%, concomitantly reduced tissue remodeling by 50%, and decreased inflammatory cells (e.g. eosinophils down by 54%)/inflammatory factors (e.g. sCD23 down by 50%)/T_H_2 cytokines (e.g. IL-5 down by 70%)/ADAM8-positive eosinophils (down by 60%) in the lung. We further verified that BK-1361 specifically targets ADAM8 *in vivo* as the peptide caused significantly reduced levels of soluble CD23 in wild-type but not in ADAM8-deficient mice. These findings suggest that BK-1361 blocks ADAM8-dependent asthma effects *in vivo* by inhibiting infiltration of eosinophils and T_H_2 lymphocytes, thus leading to reduction of T_H_2-mediated inflammation, tissue remodeling and bronchial hyperresponsiveness. Taken together, pharmacological ADAM8 inhibition appears as promising novel therapeutic strategy for the treatment of asthma.

Asthma is a chronic pulmonary disease characterized by pathologic bronchial hyperresponsiveness (BHR), airway inflammation and airway tissue remodeling^1^,^2^,^3^. Whilst its aetiology is still unclear, asthma is treated with inhaled/oral bronchodilators and/or corticosteroids to mitigate the symptoms such as BHR and airway inflammation. However, these therapeutic strategies are not only limited in efficacy beyond the provision of symptomatic relief, but also known for undesirable side effects, particularly with long-term use of steroids, as well as a high incidence of drug-resistance^4^. Therefore, there is a pressing need to develop new therapies that may overcome the shortcomings of the currently available treatment modalities.

One potential alternative therapeutic approach is to target asthma-associated proteins as suggested by the DNA microarray analysis for whole-lung RNA of allergen-challenged mice^5^. From this perspective, a disintegrin and metalloprotease 8 (ADAM8) is of particular interest since it has been identified as a signature gene associated with moderate to severe asthma. In sputum and endobronchial biopsies obtained from moderate and severe asthmatic patients, and in lungs of experimental murine asthma model, ADAM8 is more abundant. The increase in ADAM8 expression occurs in bronchial epithelium, perivascular and peribronchial immune cells such as eosinophils, macrophages, monocytes, dendritic cells (DCs) and B lymphocytes^6^,^7^,^8^,^9^,^10^,^11^,^12^,^13^,^14^. Moreover, it has been shown that for full manifestation of asthma, ADAM8 is required to be expressed in both hematopoietic and non-hematopoietic cells, however, loss of ADAM8 on T cells alone is sufficient to decrease asthma response significantly^11^, demonstrating an important role of ADAM8 in asthma pathogenesis. Furthermore, ADAM8 is known to be highly inducible by inflammatory stimuli such as interferon (IFN)-γ, lipopolysaccharide (LPS) and tumor necrosis factor (TNF)-α, suggesting a regulatory role in inflammation^15^,^16^. Moreover, there is evidence that ADAM8 can act as a sheddase for various cytokines and molecules involved in cell adhesion and extra cellular matrix (ECM) attachment such as CD23, L-selectin, vascular cell adhesion molecule-1 (VCAM-1), neural cell adhesion molecule close homologue of L1 (CHL1), pro-TNF and fibronectin. Thus, activities of ADAM8 causing the release of inflammatory cytokines, adhesion molecules and degradation of ECM proteins, are all instrumental for immune cell recruitment and the subsequent immune response in asthma^17^,^18^,^19^,^20^,^21^,^22^. More importantly, loss of ADAM8 in *ADAM8*-knockout mice, whilst showing no effect on the fertility and development of the mice, provides moderate protection from BHR and airway inflammation in ovalbumin (OVA)-induced experimental murine model of asthma, suggesting that transient and specific inhibition of ADAM8 function *in vivo* might be a potential method for treating asthma without major side effects^11^,^23^.

Thus, the pathological induction of ADAM8 under inflammatory conditions on the one hand and its dispensability for homoeostasis on the other hand favors ADAM8 as a promising therapeutic target. To test this hypothesis, the current study examined the therapeutic effect of a specific peptide inhibitor of ADAM8, named BK-1361, on an established, OVA-induced murine model of asthma. This peptide has recently been demonstrated to specifically inhibit autocatalytic activation and cellular shedding ability of ADAM8 through blocking the disintegrin/cysteine rich domain-mediated ADAM8 multimerisation without interfering with the catalytic activities of other proteases including ADAM9, 10, 12, 17, and matrix metalloproteinase (MMP)-2, -9 and -14, respectively^24^. Furthermore, the administration of BK-1361 peptide at 1, 5, 10 μg/g body weight (abbreviated as μg for the unit of dosage, thereafter) either in a single or repeated doses causes no toxic effect in mice as no abnormalities at histological level are observed, but the inhibition of ADAM8 by BK-1361 in a pancreatic ductal adenocarcinoma (PDAC) mouse model causes a significant therapeutic effect by reducing the grade of cell migration and infiltration, and thus reduced metastasis^24^. These findings prompted us to evaluate the potential of BK-1361 as an alternative therapeutic agent in asthma. We found that treatment with BK-1361 could effectively attenuate BHR, bronchial inflammation and airway wall tissue remodeling in OVA-sensitized and challenged Balb/c mice, by specifically targeting ADAM8 and thus suppressing the expression of important T_H_2 cytokines *in vivo*. These findings, therefore, provide the first evidence that targeting ADAM8 with a specific inhibitor such as BK-1361 may have great potential as a novel therapy for asthma treatment, and warrants further evaluation.

## Results

### ADAM8 inhibition with BK-1361 reduces the extent of airway responsiveness

**Figure 1:**
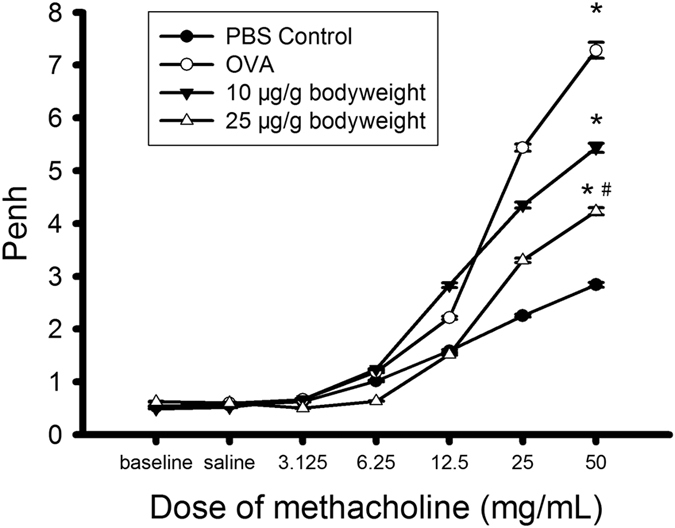
Airway resistance (Penh) of Balb/c mice versus methacholine (MCh) concentration. Penh was measured by using whole-body plethysmography when mice were stimulated with MCh at increasing doses. In all cases, Penh increased with increasing MCh. However, Penh of mice (OVA, open circles), as compared to non-sensitized mice (Control, solid circles) increased to a much greater extent in response to increasing dose of MCh stimulation, indicating bronchial hyperresponsivess (BHR). This large response of Penh to MCh in OVA-sensitized and challenged mice was attenuated dose dependently by treatment with BK-1361 (OVA, open circles versus 10 μg, solid triangles, and 25 μg, open triangles, respectively). There are statistical significances between OVA and Control, OVA and 25 μg, Control and 10 μg, Control and 25 μg, 10 μg and 25 μg (P < 0.05). Data are representative of two independent experiments with n = 8–10 age-matched female mice for each group. Results are expressed as mean ± SEM. *P < 0.05 versus control. ^#^P < 0.05 versus OVA.

Methacholine (MCh) challenge caused dose-dependent increase of enhanced pause (Penh) measured by plethysmography in all cases, but the extent to which Penh increased in response to same dose of MCh stimulation was variable in the different treatment groups (Fig. 1). In the absence of BK-1361, OVA-sensitized and challenged mice displayed significantly higher Penh, indicating BHR compared to non-sensitized mice (*OVA* vs. *control*, p < 0.05). However, the OVA-sensitized & challenged mice treated with a dose of 25 μg BK-1361 had only a moderate response to MCh compared to their counterparts without BK-1361 treatment (Penh = 4.24 ± 0.06 in *25 μg* vs. 7.28 ± 0.15 in *OVA* at 50 mg/mL MCh, p < 0.05), indicating desirable control of BHR by BK-1361. The effect of BK-1361 at 10 μg dosage was similar, but not as evident as that at 25 μg (Penh = 5.43 ± 0.08 in *10 μg* vs. 7.28 ± 0.15 in *OVA* at 50 mg/mL MCh, p < 0.05).

### BK-1361 reduces bronchial tissue remodeling

**Figure 2:**
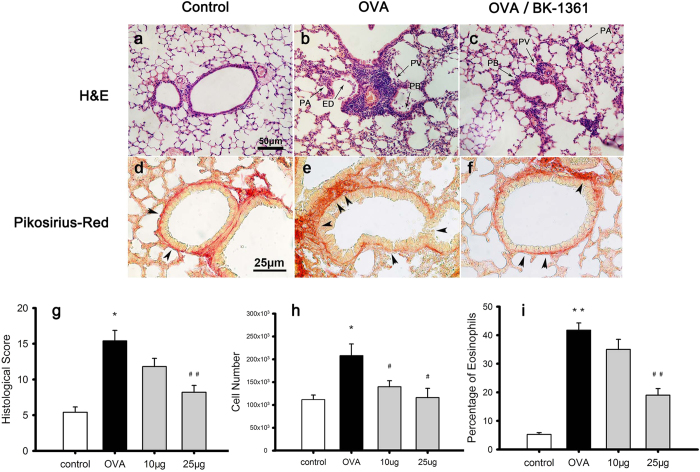
Representative micrograph images of lung sections and the quantitative histological scores, as well as total cell number and eosinophil percentage in BALF. Lung tissue sections were stained by hematoxylin & eosin (H&E) and Pikosirius-Red as shown in panel a–c, and panel d–f, respectively. Panel a and d, b and e, c and f correspond to lung sections from *control, OVA* and *OVA/BK-1361* (25 μg/g bodyweight) groups, respectively. The bar in panel a–c equals 50 μm, whereas the bar in panel d-f equals 25 μm. The arrows in panel a–c point to perivascular infiltration (PV), peribronchial infiltration (PB), parenchymal infiltration (PA), epithelial damage (ED), respectively. The arrows in panel d–f point to collagen and fibrin deposition in the bronchiole basement membranes. Panel g displays the quantified histological scores considering the extent of PV, PB, PA and ED in the lung tissue sections from different groups of mice, with score 0 indicating no sign of disease, score 5 indicating profound inflammation for each index, thus 20 being the maximum. Panels h and i display the total cell counts number and the percentage of eosinophils in bronchoalveolar lavage fluid (BALF) from different groups of mice respectively. Data in panel g are representative of two independent experiments with n = 7–9 age-matched female mice for each group, and data in panel h and i are representative of n = 5–6 female mice per group. Results are expressed as mean ± SEM. *P < 0.05, **P < 0.005 versus control. ^#^P < 0.05, ^##^P < 0.005 versus OVA.

Next, we assessed the effect of BK-1361 on bronchial tissue remodeling (Fig. 2). Representative histological lung sections stained with either hematoxylin & eosin (H&E) or Pikosirius-Red for each group are shown in Fig. 2a–f, respectively, and their quantitative scores for extent of tissue remodeling are summarized in Fig. 2g. Compared to the *control* group (Fig. 2a,d), sensitization and challenge with OVA induced significant tissue inflammation and remodeling in the mouse bronchial airways, characterized by severe perivascular (PV), peribronchial (PB), and parenchymal (PA) infiltration of inflammatory cells together with bronchial epithelial damage (ED) (Fig. 2b vs. a). Furthermore, the group exhibited severe hyperplasia of collagen and fibrin in the bronchiolar basement membrane (Fig. 2e vs. d). This resulted in an overall high total histological score of 15.40 ± 1.47 for this group as compared to 5.40 ± 0.75 for the non-sensitized mice (*OVA* vs. *control*, p < 0.05 in Fig. 2g). OVA-sensitized & challenged mice treated with BK-1361 at 25 μg had less severe PV and PB (Fig. 2c vs. b), and displayed a reduced hyperplasia of the basement membrane and an almost complete absence of ED (Fig. 2f vs. e), resulting in a decreased histological score of 8.20 ± 0.97 as compared to that for the *OVA* group (*25 μg* vs. *OVA*, p < 0.005 in Fig. 2g). However, the treatment of BK-1361 at 10 μg showed little effect on tissue remodeling (*10 μg* vs. *OVA* in Fig. 2g, lung section images not shown).

### BK-1361 reduces bronchial inflammation

The total cell counts and differential cell counts of inflammatory cells in bronchoalveolar lavage fluid (BALF) were quantified to evaluate the therapeutic effect of BK-1361 on the invasion of inflammatory cells into airways. As shown in Fig. 2h, the OVA-sensitized & challenged mice exhibited approximately 50% higher total cell counts in BALF as compared to their non-sensitized counterparts (*OVA* vs. *control*, p < 0.05), indicating increased infiltration of inflammatory cells in the murine asthma model, as mirrored by the human clinical presentation. However, significant decreases in cell number in BALF were observed after administration of BK-1361 at both 10 μg and 25 μg to OVA-sensitized & challenged mice (*10 μg* and *25 μg* vs. *OVA*, p < 0.05), suggesting that treatment with BK-1361 reduced the invasion of inflammatory cells into the airways.

For differential cell counts, OVA-sensitized & challenged mice showed a dramatically increased proportion of eosinophils in BALF when compared to the non-sensitized mice (*OVA* vs. *control*, p < 0.005), as shown in Fig. 2i and observed in previous studies^6^,^25^,^26^. This increase of eosinophils proportion, however, was significantly reduced by administration of BK-1361 at 25 μg (*25 μg* vs. *OVA*, p < 0.005), suggesting an inhibition of prominently eosinophilic infiltration in airways due to BK-1361. Interestingly, as shown in Table 1, it appeared that the proportion of neutrophils or lymphocytes in BALF was not affected by OVA-sensitization and BK-1361 treatment. In contrast, the proportion of macrophages in BALF was reduced in OVA-sensitized & challenged mice, but then increased when the mice were subsequently treated with BK-1361, even though the absolute number of macrophages in BALF was lowered by BK-1361 treatment (data not shown).

### BK-1361 reduces the recruitment of ADAM8-positive eosinophils

**Figure 3:**
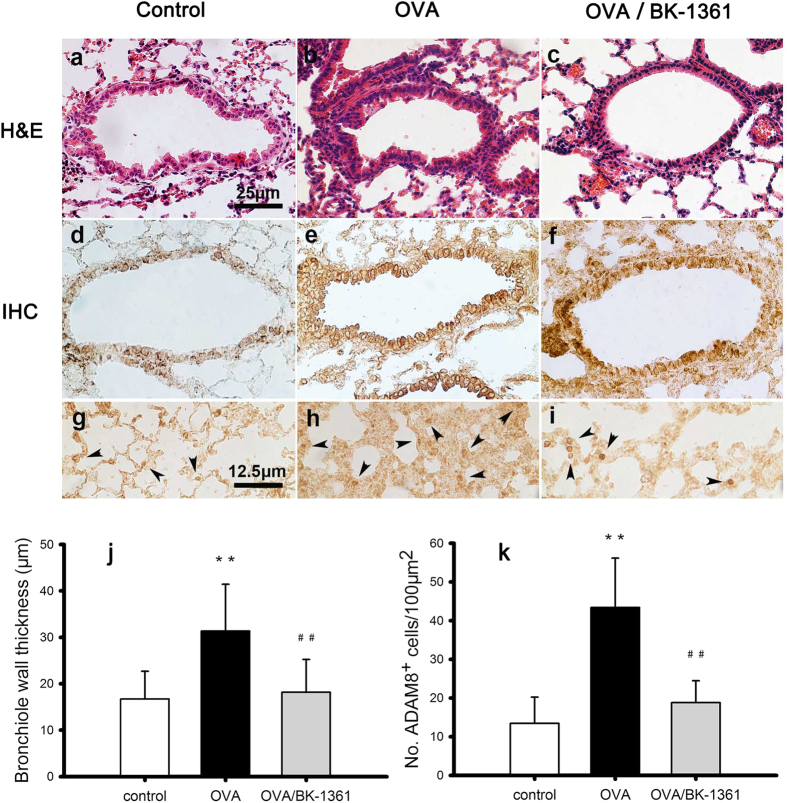
Expression of ADAM8 in lung tissue sections from different groups of Balb/c mice. Lung tissue sections were stained by hematoxylin & eosin (H&E) shown in panel a–c, and immunohistochemistry (IHC) shown in panel d–f to visualize bronchiolar structure and ADAM8 expression, respectively. Panel g-i display zoom-in images corresponding to panel d–f, with arrows pointing to ADAM8-positive eosinophils. The bar in panel a-f equals 25 μm, whereas the bar in panel g-i equals 12.5 μm. The left, middle, and right column of images represents lung tissue sections from *control* group (panel a, d, g), *OVA* group (panel b, e, h), and *OVA/BK-1361* group (panel c, f, i), respectively. Note the OVA-induced thickening of bronchiolar epithelium compared to control (panel b vs. a), which was reduced by BK-1361 treatment (panel c vs. b), and the OVA-induced fibrotic changes (panel h vs. g), which was also reduced by BK-1361 treatment (panel i vs. h). ADAM8-positive eosinophils (ADAM8^*+*^cells) appeared mainly in *OVA* group (panel h vs. g), and became much less abundant after BK-1361 treatment (panel i vs. h). Panel j, k displays quantified bronchial wall thickness, and number of ADAM8^*+*^cells, respectively. Data are representative of two independent experiments with n = 30–40 counts from 3–4 age-matched female mice per group. Result are expressed as mean ± SD. **P < 0.005 versus control. ^##^P < 0.005 versus OVA.

To understand the *in situ* effects of BK-1361 on the expression and distribution of ADAM8 further, we examined mouse lung sections for the presence of ADAM8-positive eosinophils using immunohistochemistry (IHC) since eosinophils are known to express ADAM8 protein^24^. As shown in Fig. 3, we found that in lung sections from OVA-sensitized & challenged mice, when compared to their non-sensitized counterparts, there were not only more eosinophils and bronchial epithelial cells, as well as increased interstitial edema (Fig. 3b/e vs. a/d), but also more detectable ADAM8-positive eosinophils (Fig. 3e/h vs. d/g). In contrast, when the OVA-sensitized mice were treated with BK-1361 at 25 μg dosage, not only the fibrosis thickening of bronchiolar epithelium was reduced, but also the number of detectable ADAM8-positive eosinophils in the lung sections (Fig. 3c/f/i vs. b/e/h). The quantitative estimates of bronchiole wall thickness and the number of ADAM-positive eosinophils observed in the above are summarized in Fig. 3j and k respectively. All these results suggest that inhibition of ADAM8 by BK-1361 reduces migration of ADAM8-positive cells into the airways.

### BK-1361 reduces mRNA expression of key T_H_2 cytokines

**Figure 4:**
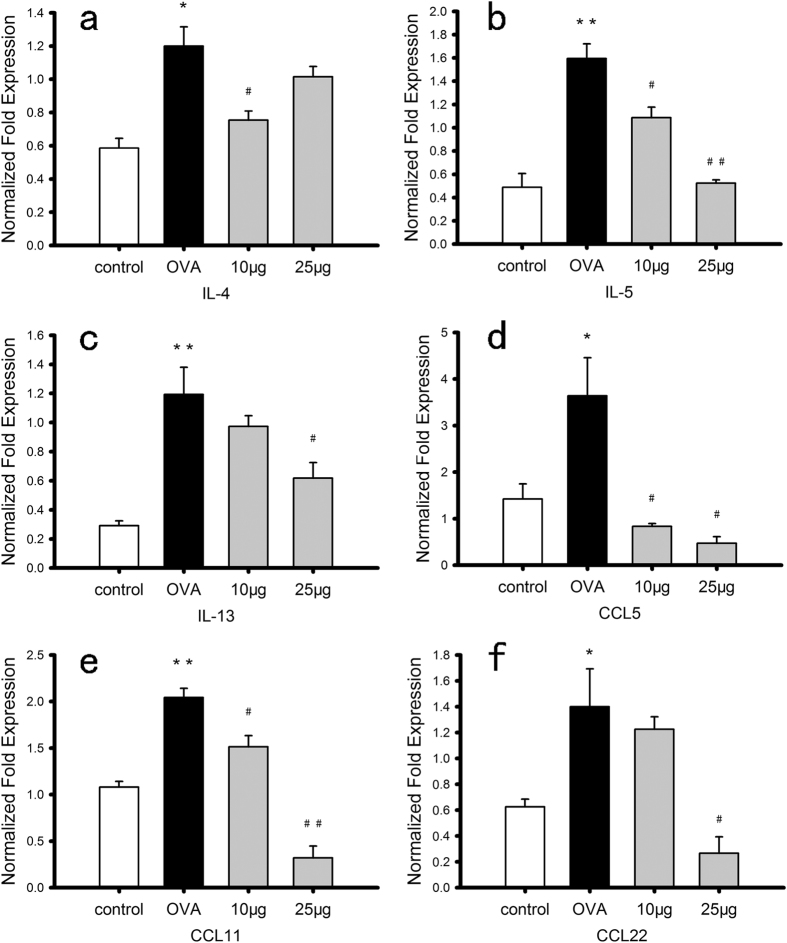
mRNA expression of T_H_2 cytokines in total lung tissue from different groups of Balb/c mice. The level of mRNA expression was quantified by RT-PCR with detection of *β*-actin and glyceraldehyde-3-phosphate dehydrogenase (GAPDH) as endogenous controls. Panel a–f display the normalized fold expression of IL-4, IL-5, IL-13, CCL5, CCL11, and CCL22, respectively, for mice of control group (*control*), OVA-sensitized and challenged group (*OVA*), and group of OVA-sensitization with BK-1361 treatment at either dose of 10 μg (*10 μg*) or 25 μg (*25 μg*). Data are representative of two independent experiments with n = 3–4 age-matched female mice per group. Result are expressed as mean ± SEM. *P < 0.05, **P < 0.005 versus control. ^#^P < 0.05, ^##^P < 0.005 versus OVA.

Asthma is also thought to be induced by T_H_2 cytokines, and we thus explored the mechanism underlying the therapeutic effect of BK-1361 by assessing the quantitative mRNA expression of several key T_H_2 cytokines associated with pathogenesis of asthma, including interleukins (IL)-4, IL-5, IL-13, chemokine (C-C motif) ligand (CCL)5, CCL11 and CCL22. In agreement with previous observations^6^,^11^, the mRNA expression of all these cytokines in lungs of OVA-sensitized & challenged mice were significantly increased compared to non-sensitized mice (p < 0.05), and reduced in the BK-1361 treated group (Fig. 4a–f). More specifically, treatment with BK-1361 at 25 μg resulted in significant decrease in expression of IL-5, IL-13 and CCL5, CCL11 and CCL22 compared to OVA-sensitized & challenged mice (p < 0.05). Remarkably, treatment with BK-1361 of OVA-sensitized & challenged mice appeared to reduce the expression of CCL5, CCL11 and CCL22 to even below that of non-sensitized mice. Treatment with BK-1361 at 10 μg reduced the expression of IL-4, IL-5, CCL5 and CCL11 in similar fashion to that at 25 μg. The decreased expression of T_H_2 cytokines was also correlated with the decreased infiltration of inflammatory cells as described above, suggesting a beneficial effect of BK-1361 on T_H_2 cytokine-mediated airway inflammation.

### BK-1361 reduces the levels of soluble CD23 and ADAM8 in lungs of OVA-sensitized & challenged mice

**Figure 5:**
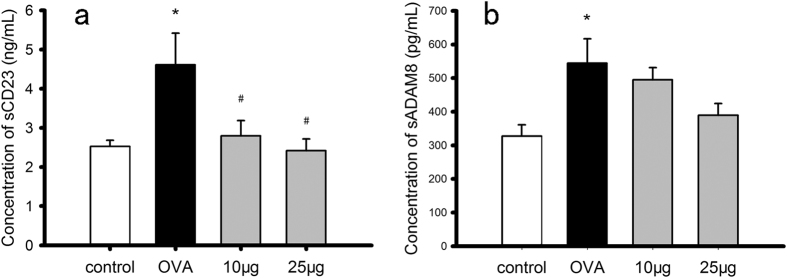
Concentration of soluble CD23 (sCD23) and soluble ADAM8 (sADAM8) in soluble protein fraction from different groups of Balb/c mice. The concentration of sCD23 and sADAM8 was quantified by ELISA, and is displayed in Panel a and b respectively, for mice of control group (*control*), OVA-sensitized and challenged group (*OVA*), and group of OVA-sensitization with BK-1361 treatment at either dose of 10 μg (*10 μg*) or 25 μg (*25 μg*). Each experiment was performed in duplicate or triplicate with n = 5–6 samples per group. Result are expressed as mean ± SEM, *P < 0.05.

CD23, the low affinity immunoglobin E (IgE) receptor, has been identified as a substrate of ADAM8 *in vitro*^21^. Previously, we have demonstrated that BK-1361 inhibits shedding of mouse CD23 in a cell-based shedding assay^24^. Here, we further investigated whether *in vivo* BK-1361 could either affect the shedding of CD23 through inhibiting the catalytic activity and endogenous processing of ADAM8, or reduce the CD23 and ADAM8 expression through decreasing the overall inflammation by assessing the expression of both soluble CD23 (sCD23, produced by CD23 shedding) and soluble ADAM8 (sADAM8, produced by endogenous processing of ADAM8) in lungs. The concentrations of sCD23 and sADAM8 were determined by ELISA in centrifuged supernatants of homogenized lung extracts. As shown in Fig. 5, when mice were OVA-sensitized and challenged, the concentrations of sCD23 and sADAM8 were both increased (*OVA* vs*. control*, p < 0.05, in both Fig. 5a,b), which is in agreement with previous findings^6^,^12^. However, when OVA-sensitized & challenged mice were treated with BK-1361, the concentration of sCD23 was significantly decreased (*OVA* vs. either *10 μg* or *25 μg*, p < 0.05, in Fig. 5a), whilst that of sADAM8 remained similar (*OVA* vs. either *10 μg* or *25 μg*, p > 0.05, in Fig. 5b), indicating that the inhibition of ADAM8 by BK-1361 *in vivo* resulted in reduced shedding of CD23.

### BK-1361 reduces CD23 shedding in wild-type but not in ADAM8-deficient mice

**Figure 6:**
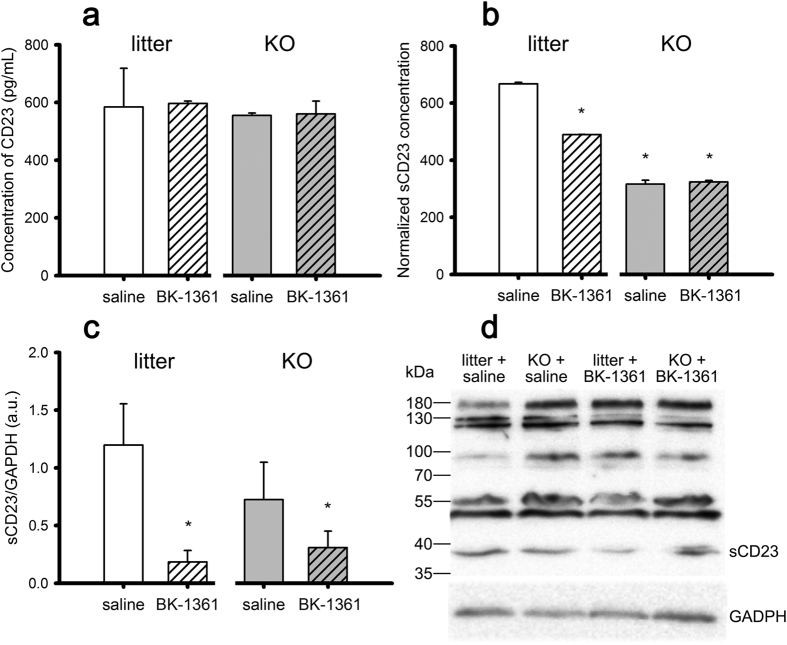
ELISA and Western Blot (WB) analysis of CD23 in wild-type (“litter”) and ADAM8-deficient (“KO”) mice after treatment with either saline or BK-1361 at 10 μg via intraperitoneal injection (i.p.) daily for 7 days. Total CD23 in lung tissue lysates and soluble CD23 (sCD23) in soluble protein fraction detected via ELISA are presented in panel a, and b respectively. The WB analysis results of sCD23 in lung tissue lysates and the representative image ofWB bands of CD23 are displayed in panel c, and d, respectively. Note the band for sCD23 around 38 kDa. For the quantification, values are derived from n = 3 (wild-type) and n = 4 (ADAM8-deficient) and are given as mean ± SEM. *p < 0.05 versus litter treated with saline.

To further verify that the decreased sCD23 expression was due to a dampened CD23 cleavage *in vivo* provided by the specific inhibitory effect of BK-1361 on ADAM8, we administered BK-1361 in ADAM8-deficient and wild-type mice at a dosage of 10 μg intraperitoneally (i.p.) daily for one week, respectively, and examined the total CD23 in lung tissues lysates and sCD23 in the soluble protein fraction by ELISA, whilst the sCD23 protein levels in lysates were also examined by western blot. As shown in Fig. 6a, there is not significant difference in the concentrations of total CD23 in the lysates from wild-type mice with or without treatment of BK-1361. In addition, ADAM8-deficient mice showed only a mild decrease in total CD23 concentrations as compare to wild-type mice, and treatment with BK-1361 did not result in further decrease (Fig. 6a). These data suggest that the total CD23 expression is independent of ADAM8 activity *in vivo*. However, the sCD23 level in both the soluble protein fractions (Fig. 6b) and the lung tissue lysates (summarized in Fig. 6c, specific band of ~38 kDa as shown in Fig. 6d) appeared to be reduced considerably in the ADAM8-deficient mice compared to the wild-type mice. Furthermore, the application of BK-1361 for one week resulted in significantly reduced sCD23 levels in both soluble protein fractions (Fig. 6b) and the lung tissue lysates (Fig. 6c) from the wild-type mice compared to the control group that received saline only (*litter + BK-1361* vs. *litter + saline*, p < 0.05, in Fig. 6b/c). By contrast, the treatment of BK-1361 for one week failed to cause further decrease of the sCD23 levels either in the soluble protein fractions or lung tissue lysates (Fig. 6b/c) from ADAM8-deficient mice. These data suggest that ADAM8 is involved in shedding of CD23 *in vivo*, and more importantly, BK-1361 reduces shedding of CD23 *in vivo* through inhibition of ADAM8.

## Discussion

In this study, we assessed the potential therapeutic effect of BK-1361, a specific peptide inhibitor of ADAM8, in treatment of the OVA-induced murine model of asthma. For this purpose, BK-1361 was administered to OVA-sensitized and challenged Balb/c mice at a dose of 10 μg or 25 μg during sensitization. These doses were predetermined according to preliminary experiments as described in the Methods.

We found that treatment with BK-1361 at 25 μg significantly reduced the extent of MCh-induced increase in Penh in OVA-sensitized & challenged mice, whilst at 10 μg the reduction was only moderate (Fig. 1). This result indicates an attenuated BHR in OVA-sensitized & challenged mice when ADAM8 activity was blocked by BK-1361, which is in agreement with previous observations in ADAM8-deficient mice^11^,^25^. To elucidate the mechanism underlying the attenuated BHR mediated by BK-1361, we further assessed the severity of airway inflammation and airway remodeling in OVA-sensitized & challenged mice after treatment of BK-1361.

The total cell number count (Fig. 2h), the percentage of eosinophils in BALF (Fig. 2i and Table 1), the severity of PV, PB, ED and hyperplasia in airway smooth muscle (ASM) layer and basement membrane, fibrosis in lung tissue and thickening of bronchiolar epithelium (Fig. 2c,f, also see Figure S7) were all decreased after administration of BK-1361 at the 25 μg dose, suggesting that the severity of eosinophilic inflammation and tissue remodeling in airways were reduced when ADAM8 activity was inhibited by BK-1361. This is in agreement with previous observations with ADAM8 either being knocked out or inhibited by antibody treatment in murine models^11^,^25^. This attenuated asthma response is likely to stem from the impaired recruitment of inflammatory cells, such as eosinophils, into the airways.

As reported before, it is essential in the elicitation and development of an asthma response that inflammatory cells migrate from the surrounding vasculature into airway mucosa and bronchoalveolar space^27^,^28^,^29^. This leads to exaggerated recruitment of these cells to bronchial and perivascular sites, which aggravates airway inflammation and subsequently contributes to the remodeling process of airway structures including functional alterations of epithelial cells, fibroblasts and ASM cells, ultimately leading to enhanced BHR and airway constriction^30^,^31^,^32^. Previous studies have also demonstrated a functional association of ADAM8 in the migration of various inflammatory cells and ECM degradation, indicating an important regulatory role of ADAM8 in the infiltration and recruitment of these cells to sites of airway inflammation^11^,^17^,^22^,^33^,^34^. Therefore, ADAM8 inhibition by BK-1361 provides a promising opportunity for the treatment of asthma.

In agreement with this notion, our results seem to imply that BK-1361 may treat asthma through a mechanism of directly inhibiting ADAM8 activity on inflammatory cells, especially eosinophils, and thus reducing their ability to migrate into the bronchoalveolar space, consistent with its role in reducing cellular invasion in a PDAC mouse model^24^.

To further support this proposed mechanism underlying the therapeutic effect of BK-1361, we detected mRNA expression of several typical T_H_2 cytokines including IL-4, -5, -13 and CCL5, 11, 22 in lung tissues of mice. These cytokines are known to activate inflammatory and residential effector pathways in asthma both directly and indirectly^35^,^36^,^37^. Specifically, both IL-4 and IL-13 regulate differentiation of T_H_0 cells to develop a T_H_2 phenotype and stimulate B cells to produce IgE. IL-5 promotes proliferation of eosinophils, which together with IgE are both important components in development of allergic inflammation and BHR^1^,^38^. CCL22 is a macrophage-derived chemokine and plays an important role in chronic allergic inflammation^39^. CCL11 is mainly secreted by eosinophils^40^,^41^, and CCL5 is thought to be involved in pathogenesis of late-onset asthma^42^. Accordingly, the expression levels of these typical T_H_2 cytokines could offer an overall reflection of severity of T_H_2 cytokines-mediated inflammation. Interestingly, ADAM8 has been reported as an IL-4 and IL-13 induced gene and can be regulated through different signaling pathway in experimental asthma^6^,^26^. In addition, decreased expression of CCL11 and CCL22 in OVA-sensitized & challenged mice with *Adam8* knockout or knocked down have also been demonstrated before^25^, indicating that ADAM8 is involved in the T_H_2 cytokines-mediated inflammation. In agreement with this view, our results showed significantly decreased expression levels of IL-5, CCL5, CCL11 and CCL22, and moderately attenuated expression of IL-4 and IL-13 in OVA-sensitized & challenged mice that were treated with BK-1361 at either 10 μg or 25 μg as compared to their untreated counterparts (Fig. 4). These results suggest a desirable control of T_H_2 cytokines-mediated inflammation in OVA-sensitized & challenged mice by inhibition of ADAM8 function *in vivo*, which may also be induced by the impaired recruitment of T_H_2 cells

In addition, ADAM8 has been shown *in vitro* to shed CD23 that binds to IgE and eventually results in immune response^43^. Expression of CD23 is decreased in IL-4- and IL-13-deficient mice, indicating a synergistic mode between CD23 and IL-4/IL-13^38^. In agreement with these observations, our results showed decreased sCD23 level in the soluble protein fractions from lung tissues of OVA-sensitized & challenged mice after treatment with BK-1361 at either 10 μg or 25 μg (Fig. 5a), associated with a decreased mRNA expression of IL-4 and IL-13 as described above. This decreased sCD23 level might be a combinatorial result of an overall decreased inflammatory cells invasion mediated by BK-1361, as well as BK-1361-dependent inhibition of catalytic ability of ADAM8 to cleave CD23 *in vivo*. However, BK-1361 appeared to have little effect on the concentration of sADAM8 (Fig. 5b), which may also be reasonable considering that BK-1361 inhibits substrate shedding but not the expression of ADAM8 *in vivo*.

Furthermore, BK-1361 is known to specifically target ADAM8 *in vitro* as observed in an ADAM8-dependent cell migration assay^24^. As shown in Fig. 6b/c, BK-1361 significantly reduced the sCD23 level in the presence of ADAM8 in wild-type mice, however, when ADAM8 was lacking in ADAM8-deficient mice, the treatment of BK-1361 either had little effect on the sCD23 level in the soluble protein fraction (Fig. 6b, detected by ELISA), or resulted in a only mild decrease in sCD23 levels in lung tissue lysates (Fig. 6c, detected by western blot). This suggests that BK-1361 also targets ADAM8 *in vivo* to at least in part reduce the shedding of sCD23 according to the latter’s dependence on the presence of ADAM8. Although ADAM8 is not the only ADAM protease that serves as a sheddase for CD23 *in vitro*, and it is even controversial whether ADAM8 sheds CD23 *in vivo*^44^, the observed decrease in CD23 shedding in wild-type mice could only result from the effect of BK-1361 on ADAM8 but not on other ADAM proteases such as ADAM10 as described before^24^. However, it is still possible that inhibition of CD23 shedding can be either direct or indirect (i.e. by inhibiting activation of ADAM10 mediated by ADAM8). Interestingly, the ADAM8-deficient mice showed no difference on total CD23 expression levels but decreased sCD23 levels compared to wild-type mice, indicating that ADAM8 might at least account for partial CD23 shedding *in vivo*.

It is worth noting that although several studies have assessed the role of ADAM8 in experimental asthma, the findings of these studies seem to be controversial^11^,^25^,^45^,^46^. This may have been caused, at least partially, by the different mouse strains used in those studies. For example, the T_H_1-skewed strains (such as C57Bl/6 strain) have congenitally reduced recruitment of eosinophils and T_H_2 cells during airway inflammation compared to T_H_2-skewed strains^3^,^47^. Here we show that in a pure T_H_2-skewed strain (Balb/c strain), ADAM8 facilitated the infiltration of eosinophils and T_H_2 cells and thus participated in the aggravation of asthma airway inflammation.

There are recognized limitations of the current study. For instance, we evaluated the therapeutic effect of BK1361 only in an acute murine model of asthma, which is more relevant to inflammatory mechanisms of the disease. For the long-term effect of BK-1361 on asthma, and particularly on tissue remodeling as a consequence of prolonged inflammation^48^, chronic murine asthma models are required in future studies. In addition, the half-life of current form of the peptide is relatively short^24^, which should be extended in order to further assess its efficacy and therapeutic dosing strategy, as well as possible clinical use. Another issue is that in the present study, Penh, an unrestrained plethysmography parameter, was used as an indicator of BHR in mice. This technique is advantageous in terms of convenience and non-invasiveness. However, it has been questioned whether measurement of Penh truly reflects the BHR as compared to the measurement of changes of airway resistance by direct technique such as force oscillatory technique (FOT)^49^,^50^. Although Penh is not considered the best choice for accurate measurement of BHR in general, this technique may still be used to estimate BHR under specific, controlled conditions. For example, it has been shown that Penh is well correlated with airway resistance only when Balb/c mice are stimulated with MCh at high dose so that the variations of nonmechanical origins are minimized^49^. Considering that we also used Balb/c mice and the highest dosage of MCh (50 mg/mL) in this study, as well as a well-established protocol to induce BHR, it is probably reasonable that the measured Penh largely reflected the airway resistance and thus was justified to quantitatively characterize BHR.

Taken together, our findings demonstrate that ADAM8 might be a novel drug target for treatment of allergic asthma, perhaps other T_H_2 cytokines-mediated inflammatory diseases as well, because inhibition of ADAM8, at least with BK-1361, could suppress asthma symptoms from BHR to airway inflammation and tissue remodeling without major side-effects in this model used.

## Materials and Methods

### Chemicals and reagents

OVA (grade V) was purchased from Sigma-Aldrich Co. (St. Louis, MO, USA); aluminum hydroxide was purchased from Kelong Tech. Co. (Chengdu, China); methacholine was purchased from Tokyo Chemical Industry Co. (Tokyo, Japan); hematoxylin & eosin stain, Wright–Giemsa stain was purchased from Jiancheng Tech. Co. (Nanjing, China); TRIzol solution was purchased from Bioteke Co. (Beijing, China); RevertAid First Strand cDNA Synthesis Kit was purchased from Thermo Fisher Scientific Inc. (Waltham, MA, USA); Oligonucleotide primers were produced by Invitrogen Life Technologies Co. (Shanghai, China); SsoFast EvaGreen Supermix was purchased from Bio-Rad Laboratories (Hercules, CA, USA); ELISA kits for soluble CD23 (sCD23) and soluble ADAM8 (sADAM8) was purchased from either Life Science Inc. (Wuhan, China)or R&D Systems (Wiesbaden, Germany); NanoDrop was purchased from PeqLab (Erlangen, Germany); the ADAM8 antibody (Cat#: orb4376) was purchased from Biorbyt (Cambridge, U.K.), CD23 antibody (abb185807) from Abcam (Cambridge, U.K.), goat anti-mouse HRP and goat anti-rabbit HRP from Jackson ImmunoResearch (West Grove, PA, USA), mouse anti-GAPDH from Thermo Fisher Scientific (Waltham, MA, USA); VECTASTAIN Elite ABC Kit was purchased from Vector Laboratories (Peterborough, U.K.).

### Experimental animals

Balb/c mice were purchased from the Laboratory Animal Centre of Third Military Medical University (Chongqing, China), maintained and bred in specific pathogen-free environment. ADAM8-deficient (*Adam8*^−/−^) and their wild-type littermates (*Adam8*^+/+^) mice were kept under SPF conditions in the mouse facility (Marburg University, Germany). Peptide injection experiments were performed with permit by the local authorities (Regierungspräsidium Giessen). All other animal experiments in this study were approved by the Institutional Animal Care and Research Advisory Committee of Third Military Medical University and performed in accordance with the guidelines of the Chinese Council on Animal Care (Approved No. 11172340, LHD).

### Allergen sensitization

Three to four weeks old Balb/c mice were randomly divided into four groups: OVA-sensitized and challenged group (abbreviated as *OVA*), OVA-sensitized and challenged with BK-1361 treatment group (abbreviated as *OVA/BK-1361*) and Control group (abbreviated as *control*), n = 8–10 for each group. Mice in both *OVA* and *OVA/BK-1361* were sensitized with OVA following standard procedures of previous studies^2^,^11^ (please see Figure S1 in the supplements for detailed protocol). Specifically, for OVA and OVA/BK-1361 group, 3–4 weeks old Balb/c mice were first acclimatized for 3 weeks, and then injected i.p. with 10 μg OVA and 100 μg aluminum hydroxide suspended in 1 mL sterile isotonic saline once a week for 3 weeks. Subsequently, the mice were exposed to aerosolized challenge of sterile isotonic saline comprising 1% OVA (1% w/v in sterile isotonic saline) for 30 min once every day for a week. Mice in the control group were treated with sterile saline using the same protocol.

### Design and administration of a peptide inhibitor specific for ADAM8 (BK-1361)

BK-1361 was described previously and has been tested for safety and efficacy in mice elsewhere^24^. In brief, homology modeling was performed based on the X-ray structure of the ADAM10 disintegrin domain^51^. An exposed sequence within the putative integrin binding loop was targeted by creating cyclic peptide variants that match the exposed sequence KDK in mouse ADAM8. Thus the optimal cyclic peptide was shown to be cyclo “RLsKDK” with “s” being a D-serine residue. The BK-1361 was synthesized by Peptide 2.0 (Chantilly, VA, USA) with a purity of >97%, and then dissolved in phosphate buffered saline (PBS) at a stock concentration of 1 mg/mL, prior to further dilutions.

Our previous study has showed that the serum concentrations of BK-1361 in mice after injecting 10 μg/g bodyweight BK-1361 via an i.p. route reached as high as ~37 ng/μL with a half life of ~34 min, indicating a good distribution of BK-1361 in the body of mice. In addition, administration of 1, 5, 10 μg/g body weight of BK-1361 either in single or repeated doses for up to 4 weeks has been suggested to have no acute or chronic toxicity effect to mice as no abnormalities at histological levels was observed^24^. Similarly, in our preliminary experiment, the mice with administration of 10, 25, 33 and 50 μg/g body weight of BK-1361 for up to 1 week showed no signs of weight loss, abnormal behavior or abnormal motor performance over study period, suggesting BK-1361 was well tolerated in Balb/c mice. Among the dosages we used, no further improvement beyond 25 μg observed (such as 33 and 50 μg/g body weight, please see Figures S2–6 in the supplements). Accordingly, for the *OVA/BK-1361* group, the peptide was administered in mice with a dose of either 10 μg/g or 25 μg/g body weight of mouse (abbreviated as *10 μg* and *25 μg* respectively). The body weight of each mouse, which varied between ~19–24 g, was determined prior to administration of BK-1361. The corresponding amount of peptide was then calculated and administered via i.p. 2 h prior to each aerosolized challenge every day for one week (please see Figure S1 in the supplements for detailed protocol). Mice in the control group were treated with the vehicle of peptide (PBS), using the same protocol.

### Assessment of airway responsiveness

The lung function was assessed 24 h after the last allergen exposure by measuring the respiratory pressure curves in response to methacholine (MCh) stimulation using a single-chamber, whole-body plethysmography set-up (EMKA Technologies, Paris, France) as previously described^52^. Briefly, the animal was placed in a sealed single chamber and exposed to either aerosolized saline (for the baseline measurement) or MCh (with increasing concentrations ranging from 3.125–50 mg/mL) for 3 min each time. Then enhanced pause (Penh) was recorded for 3 min after each nebulization as an indicator of airway responsiveness. The value of Penh was calculated by Penh = PEP/PIP × Pause (where PEP, PIP and Pause represented the peak expiratory flow, the peak inspiratory flow and the time of expiration respectively), which was thought to be an unrestrained plethysmography parameter with high correlation to the airway resistance in Balb/c mouse strain^49^.

### Assessment of bronchial inflammation

Mice were sacrificed 24 h after lung function testing, and bronchoalveolar lavage fluid (BALF) was collected by inserting a cannula into the trachea of the mice with three instillations of 0.5 mL PBS. BALF was centrifuged at 239 g for 10 min at 4 °C. Supernatants were stored at −80 °C for further analysis whilst cell pellets were resuspended in PBS to proceed with total and differential cell counts. The total cell number was determined by direct microscopy. For the differential cell counts, cells suspended in ~40–80 μL PBS was dropped upon a sterile slide, then a smear was made by streaking the cell suspension across the slide with a cover glass. The cells deposited on slides were then stained using the Wright–Giemsa method and subsequently counted by a skilled observer blinded to experimental details. The percentage of eosinophils, neutrophils, lymphocytes and macrophages were obtained respectively from each group at 400x magnification.

### Assessment of bronchial tissue remodeling

After collection of BALF, the right lungs were harvested and snap frozen in liquid nitrogen for downstream RNA extraction and lung homogenates, whereas the left lung was insufflated at constant pressure with 4% buffered formaldehyde solution. Formalin-fixed lungs were processed and embedded in paraffin, cut to 5 μm sections, and stained with hematoxylin & eosin (H&E) and Picosirius-Red respectively. Histologic inflammatory scores were obtained by an experienced histologist blinded to experimental details under 200x magnification, who took into accounts of perivascular infiltration (PV), peribronchial infiltration (PB), parenchymal infiltration (PA), and epithelial damage (ED) and gave an evaluation for each index. For each evaluation index, 0 point indicates no sign of disease whereas 5 point represents profound inflammation, thus the extent of the bronchial inflammation and associated tissue remodeling was presented as a total score point between 0 and 20 as described before^2^,^11^.

### Quantification of mRNA expression of inflammatory cytokines in lung tissue

Total RNA of lung tissues were extracted using TRIzol and reverse transcribed using RevertAid First Strand cDNA Synthesis Kit. The sequences of oligonucleotide primers for IL-4, IL-5, IL-13, CCL5, CCL11 and CCL22, as shown in Table 2, were designed based on the nucleotide sequence available in NCBI Genbank. The specificity of selected sequences was verified using the NCBI BLAST program (http://www.ncbi.nlm.nih.gov/BLAST/). The expression of mRNA for each gene was quantified using SsoFast EvaGreen Supermix, detection of *β*-actin and glyceraldehyde-3-phosphate dehydrogenase (GAPDH) were chosen as endogenous controls. All reactions were run using the CFX96 real-time PCR Detection System (Bio-Rad Laboratories, Hercules, CA, USA). Normalized fold expression of mRNA was calculated using Bio-Rad CFX Manager software 1.6 (Bio-Rad Laboratories, Hercules, CA, USA) based on the Pfaffl algorithm^53^.

### Quantification of soluble CD23 and ADAM8 expressions in lung tissue lysates

Lung tissues from sensitized and challenged mice were homogenized in ice-cold PBS, and clarified by centrifugation (10000 rpm, 10 min at 4 °C), then the supernatants were stored in liquid nitrogen for further analysis. Concentrations of the soluble CD23 (sCD23) and soluble ADAM8 (sADAM8) in the supernatants of lung extracts were measured using commercial ELISA kits according to the manufacturer’s instructions.

For analysis of BK-1361 in unchallenged mice, 4 wild-type (*Adam8*^+/+^) and 4 ADAM8-deficient (*Adam8*^−/−^) mice were injected i.p. with either 10 μg/g body weight of BK-1361 or with identical volumes of saline for one week. To test the CD23 in lysates, lung tissue was frozen in liquid nitrogen and grounded. The tissue powder was resuspended in 500 μL PBS containing 1%BSA and homogenized on ice for 45 s. To obtain the soluble protein fraction, the extract was centrifuged at 13.000 × g for 10 min at 4 °C. The soluble protein fraction was then used for ELISA analyses and protein concentrations were determined. CD23 ELISA assays were performed according to the manufacturer’s instructions and values were determined based on a standard curve. The lung tissue lysates were diluted 10 times for the test of total CD23, undiluted supernatants of lysates were used for the test of sCD23. The concentrations of sCD23 were normalized for protein concentrations (amount of BSA was subtracted from measured protein concentrations). All experiments were performed with pooled tissue from at least 3 mice and the values are derived from triplicate samples.

### Immunohistochemistry (IHC) of mouse lungs

Lung sections (5 μm thickness) from paraffin-embedded lung tissues were deparaffinized in xylene and rehydrated by an alcohol gradient. Endogenous peroxidase activity was blocked with 3% hydrogen peroxide. Sections were incubated with citrate buffer for antigen retrieval and incubated with an antibody targeting ADAM8 (1:150) followed by the ABC Vectastain method using 3,3′-diaminobenzidine (DAB) chromogen detection. Immunostained sections were counterstained with H&E and micrographs were taken using a Nikon Image Suite (Nikon, Tokyo, Japan).

### Western blot

For analysis of BK-1361 in unchallenged mice, the lung tissues from either *Adam8*^+/+ ^or *Adam8*^−/−^ mice were powdered under liquid nitrogen and subjected to lysis using PBS containing 1% Triton-X100 and 2x Complete Protease inhibitor cocktail (Roche, Germany) for 20 min on ice. 100 μg total protein per lane were subjected to a reducing 10% SDS-PAGE electrophoresis and Western blotting. Blots were blocked for 1 h with 5% milk powder in Tris buffered saline with 0.05% Tween (TBST), followed by 2 h incubation with 2 μg/mL polyclonal rabbit anti-CD23, which was detected with 27 ng/mL goat anti-rabbit HRP. After stripping, loading was controlled by incubation with 25 ng/mL mouse anti-GAPDH in 5% milk powder and 27 ng/mL goat anti-mouse HRP in TBST. Quantification of bands was performed using AIDA Image Analyzer software (Raytest, Straubenhardt, Germany).

### Statistics

The ANOVA analysis of variance followed by Tukey’s honestly significant difference analysis (Tukey’s HSD) and Student t-test was applied for statistical analysis, the value of *^/#^P < 0.05, **^/##^P < 0.005 were considered significant. Statistical analysis was performed with the OriginPro 8.5.1 SR2 (OriginLab Corporation, Northampton, MA, USA); Graphs were obtained using SigmaPlot 10.0 (Systat Software Inc., Chicago, IL, USA).

## Additional Information

**How to cite this article**: Chen, J. *et al*. A novel peptide ADAM8 inhibitor attenuates bronchial hyperresponsiveness and Th2 cytokine mediated inflammation of murine asthmatic models. *Sci. Rep.* **6**, 30451; doi: 10.1038/srep30451 (2016).

## Supplementary Material

Supplementary Information

## Figures and Tables

**Table 1:** Percentage of inflammatory cells in BALF from different groups of Balb/c mice.

%	control	OVA		10 μg		25 μg	
Eosinophils	5.25 ± 0.59	41.75 ± 2.55	^**^	35.00 ± 3.55	^**^	19.00 ± 2.30	^*, **##**^
Neutrophils	9.50 ± 1.21	12.25 ± 1.65		11.08 ± 1.19		10.08 ± 1.25	
Lymphocytes	15.75 ± 2.47	20.83 ± 1.30		19.42 ± 1.00		13.83 ± 0.77	^**#**^
Macrophages	69.50 ± 4.03	25.17 ± 1.99	^**^	34.50 ± 2.35	^**^	57.08 ± 3.66	^**##**^

Differential cell counts of four major types of inflammatory cells including eosinophil, neutrophil, lymphocyte and macrophage were obtained, and percentages of each cell type from total cell numbers was calculated for control group (*control*), OVA-sensitized and challenged group (*OVA*), and group of OVA-sensitization with BK-1361 treatment at either dose of 10 μg (*10 μg*) or 25 μg (*25 μg*) respectively. Data are representative of two independent experiments with n = 5–6 age-matched female mice for each. Results are expressed as mean ± SEM. *P < 0.05, **P < 0.005 versus control. ^#^P < 0.05, ^##^P < 0.005 versus OVA.

**Table 2:** Oligonucleotide primers used for RT-PCR.

		Sequence (5′-3′)	Length (bp)^a^	Accession No.^b^
IL-4	FW	CAAACGTCCTCACAGCAACG	166	M25892, X05253, NM_021283
	RV	AGGCATCGAAAAGCCCGAAA		
IL-5	FW	CCCTCATCCTCTTCGTTGCAT	72	X06270, X06271, NM_010558.1
	RV	CCCTTGGCTACACACTGAGTT		
IL-13	FW	CCAGCCCTCAGCCATGAAATA	111	M23504, L13028, NM_008355
	RV	CACCTTGAGTGTAACAGGCCA		
CCL5	FW	CAGCAGCAAGTGCTCCAATC	238	NM_013653
	RV	CCATTTTCCCAGGACCGAGT		
CCL11	FW	AACCCAGAGCCTAAGAACTGC	236	NM_011330
	RV	CTCGTCCCATTGTGTTCCTCA		
CCL22	FW	GCTCTCGTCCTTCTTGCTGT	208	NM_009137
	RV	GGATCGGCACAGATATCTCG		
*β*-actin	FW	AGAGGGAAATCGTGCGTGAC	138	X03672, V01217, J00691, M_007393.3
	RV	CAATAGTGATGACCTGGCCGT		
GAPDH	FW	TCACCACCATGGAGAAGGC	168	M32599, U09964, NM_008084
	RV	GCTAAGCAGTTGGTGGTGCA		

The first column lists the tested cytokines including interleukin (IL) 4, 5, 13; chemokine (C-C motif) ligand (CCL) 5, 11, 22; and references of β-actin and glyceraldehyde-3-phosphate dehydrogenase (GAPDH). The second column indicates either forward (FW) or reverse (RV) primer. The sequence of each primer, its length and accession number are listed in the 3^rd^–5^th^ column. The superscript a, and b denotes amplicon length in base pairs, and Genbank accession number of mRNA and corresponding gene (http://www.ncbi.nlm.nih.gov/), respectively.
