# Supplementary material for: A novel peptide ADAM8 inhibitor attenuates bronchial hyperresponsiveness and Th2 cytokine mediated inflammation of murine asthmatic models
Source: Sci Rep. 2016 Jul 26;6:30451. doi: 10.1038/srep30451 (PMC4960557; doi:10.1038/srep30451)
Supplement: Supplementary Information [file srep30451-s1.pdf]

Supplementary Information

A novel peptide ADAM8 inhibitor attenuates bronchial hyperresponsiveness and Th2 cytokine mediated inflammation of murine asthmatic models

Jun Chen[1, 2], Linhong Deng[1, 2*], Daniela Dreymüller[3], Xuemei Jiang[2], Jiaoyue Long[2], Yiyuan Duan[2], Yue Wang[1], Mingzhi Luo[1], Feng Lin[2], Lizhen Mao[4], Bernd Müller[5], Garrit Koller[6,7], Jörg W. Bartsch[7*]

1, Changzhou Key Laboratory of Respiratory Medical Engineering, Institute of Biomedical Engineering and Health Sciences, Changzhou University, Changzhou, Jiangsu, China.

2, Key Lab of Biorheological Science and Technology, Ministry of Education, Bioengineering College, Chongqing University, Chongqing, China.

3, Institute of Pharmacology and Toxicology, RWTH Aachen University, Wendlingweg 2, 52074 Aachen, Germany

4, Jiangsu Asialand Bio-med Technology Co. Ltd., Changzhou, Jiangsu, China.

5, Laboratory of Respiratory Cell Biology, Division of Pneumology, Philipps-University Marburg, Marburg , Germany

6, KCLDI Biomaterials, Biomimetics and Biophotonics Group , King's College London, London SE1 9RT, United Kingdom.

7, Department of Neurosurgery, Philipps-University Marburg, Baldingerstr., 35033 Marburg, Germany.

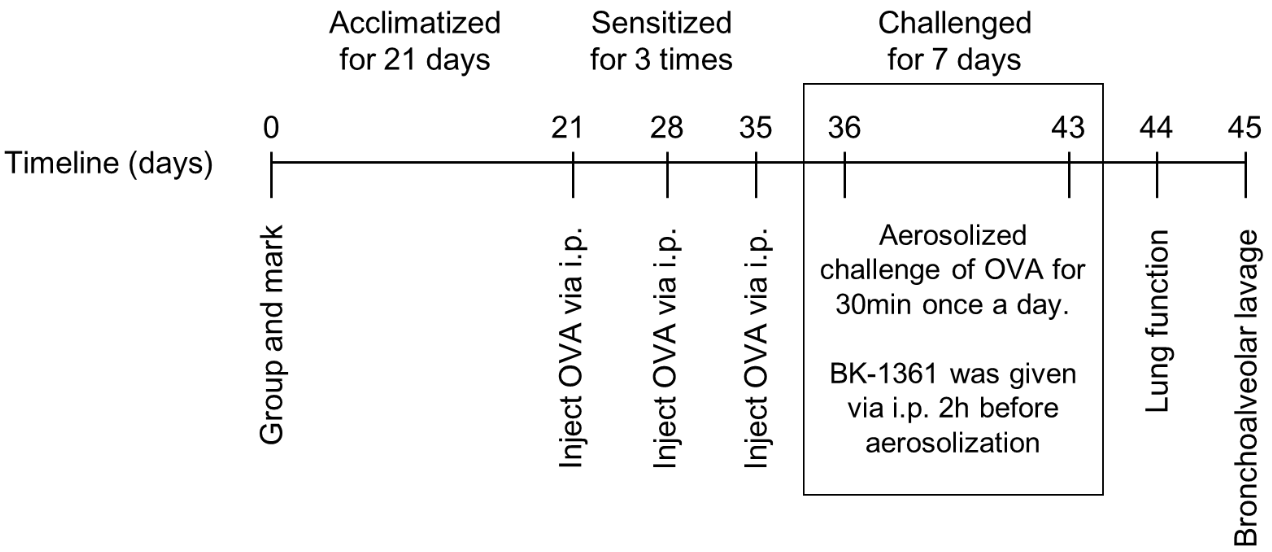

Figure S1. Protocol for OVA-sensitized and challenged mouse model of asthma.

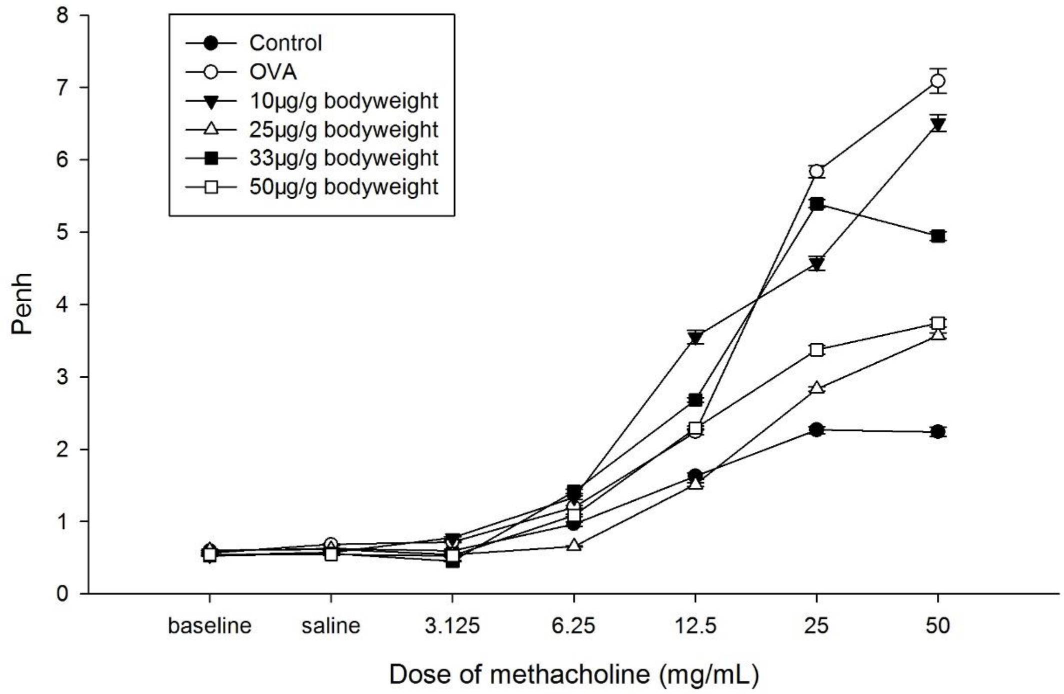

Figure S2. Airway resistance (Penh) of Balb/c mice versus methacholine (MCh) concentration. Penh was measured by using whole-body plethysmography when mice were stimulated with MCh at increasing doses. In all cases, Penh increased with increasing MCh. However, Penh of OVA-sensitized mice (*OVA,* open circles), as compared to non-sensitized mice (*Control,* solid circles) increased to a much greater extent in response to increasing dose of MCh stimulation, indicating BHR. This large response of Penh to MCh in OVA-sensitized mice was attenuated for different degrees by treatment with different dosages of BK-1361 (*OVA*, open circles versus *10μg*, solid triangles, *25μg*, open triangles, *33μg*, solid squares, and *50μg*, open squares respectively). Interestingly, as compared to the dosage of 25μg, no better effect was shown when the dosage of 33 or 50μg was given. Data are representative of two independent experiments with n>5 age-matched female mice for each group. Results are expressed as mean±SEM.

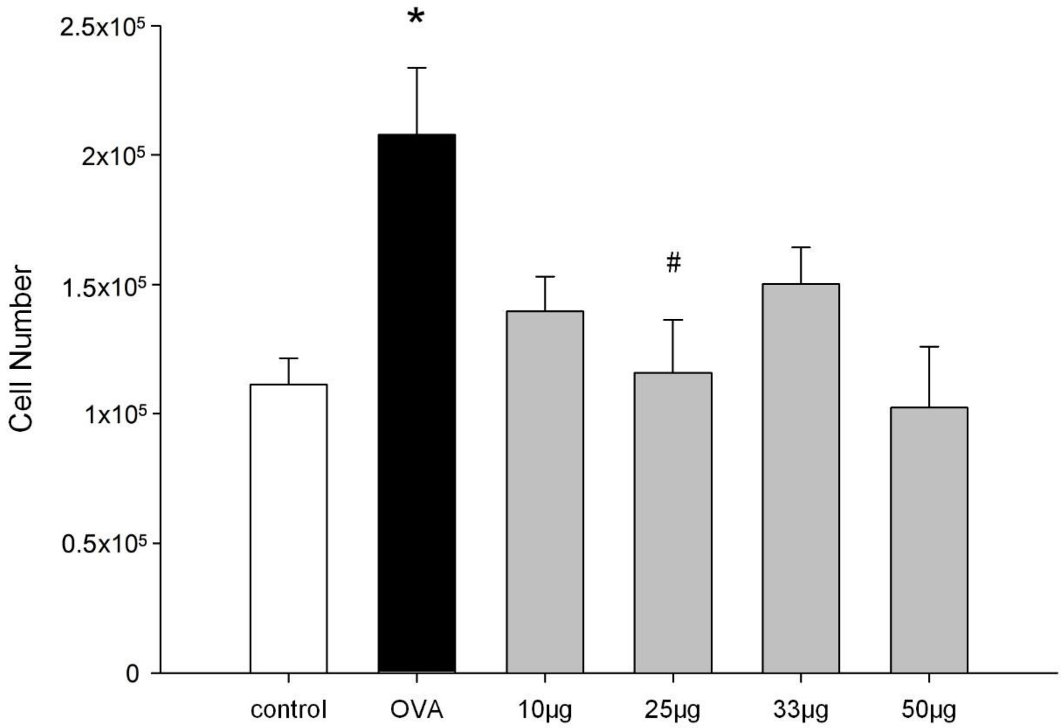

Figure S3. Total cell number in bronchoalveolar lavage fluid (BALF) from different groups of mice. The OVA-sensitized mice exhibited approximately 50% higher total cell counts in BALF as compared to their non-sensitized counterparts (*OVA* vs. *control*, $p<0.05$). However, decreased cell number in BALF were observed after treatment of BK-1361 at 25µg dose on OVA-sensitized mice (*25µg* vs. *OVA*, $p<0.05$), while the effects of BK-1361 at other doses were not significant. Data are representative of n=5–6 female mice per group. Results are expressed as mean±SEM. *$P<0.05$ versus control. #$P<0.05$ versus OVA.

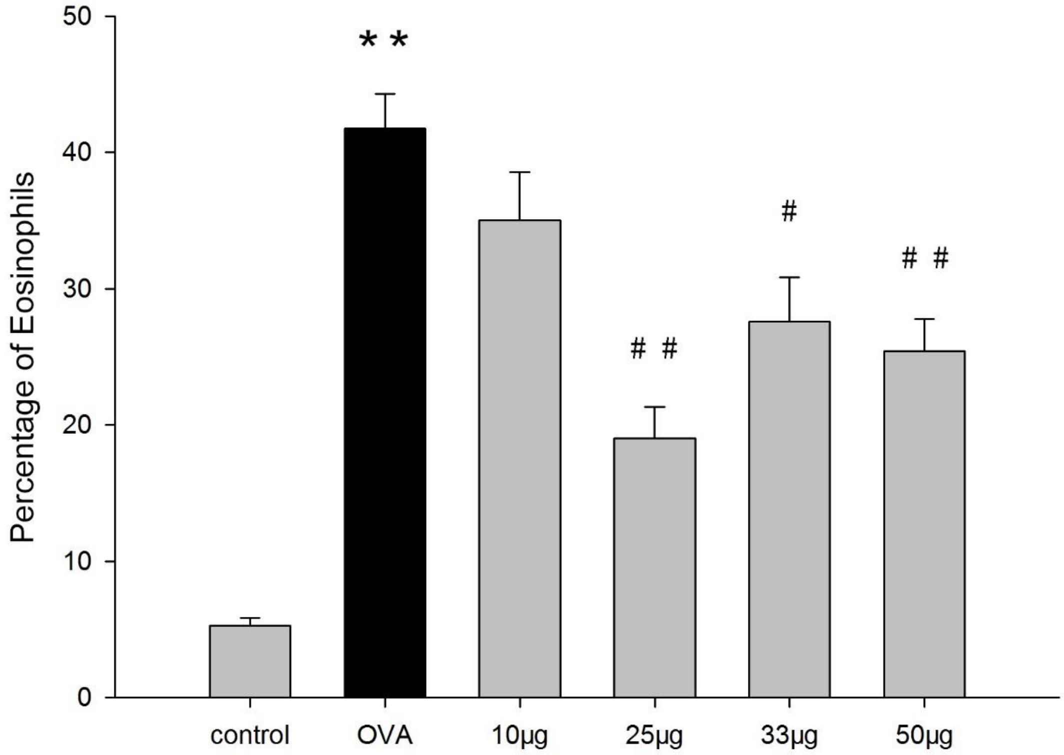

Figure S4. Percentage of eosinophils for differential cell counts in bronchoalveolar lavage fluid (BALF) from different groups of mice. The OVA-sensitized mice showed a dramatically increased percentage of eosinophils in BALF when compared to the non-sensitized mice (*OVA* vs. *control*, $p<0.005$). However, decreased percentage of eosinophils in BALF were observed after treatment of BK-1361 at 25, 33 or 50µg dose respectively on OVA-sensitized mice (*25, 33, 50µg* vs. *OVA*, $p<0.005$), while the effect of BK-1361 at 10µg dose was not significant. Interestingly, as compared to the dosage of 25µg, no better effect was shown when the dosage of 33 or 50µg was given. Data are representative of n=5–6 female mice per group. Results are expressed as mean±SEM. **$P<0.005$ versus control. #$P<0.05$, ##$P<0.005$ versus OVA.

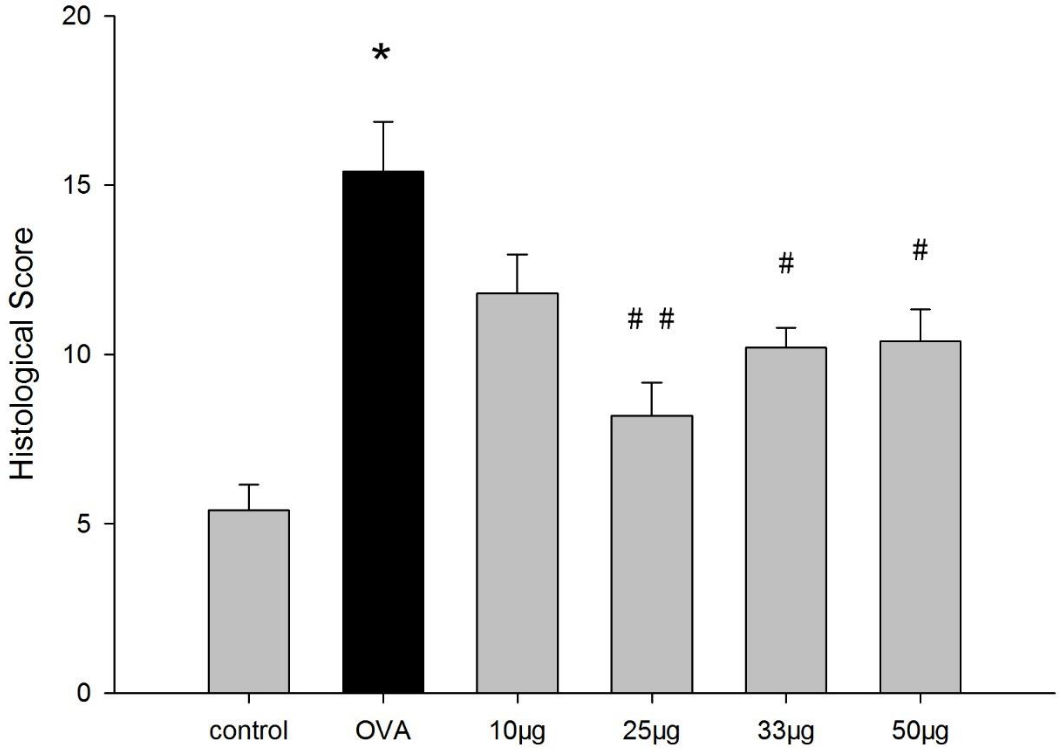

Figure S5. Quantitative histological scores of lung tissue sections from different groups of mice. The severity of perivascular infiltration (PV), peribronchial infiltration (PB), parenchymal infiltration (PA) and epithelial damage (ED) as shown in lung sections were evaluated respectively with score 0 indicating no sign of disease, 5 indicating profound inflammation, and 20 being the maximum. The OVA-sensitized mice showed increased histological score when compared to the non-sensitized mice (*OVA* vs. *control*, $p<0.05$). However, decreased histological scores were observed after treatment of BK-1361 at 25, 33 or 50μg dose respectively on OVA-sensitized mice (*25, 33, 50μg* vs. *OVA*, $p<0.005$), while the effect of BK-1361 at 10μg dose was not significant. Interestingly, as compared to the dosage of 25μg, no better effect was shown when the dosage of 33 or 50μg was given. Data are representative of two independent experiments with n=7-9 age-matched female mice for each group. Results are expressed as mean±SEM. *P<0.05 versus control. #P<0.05, ##P<0.005 versus OVA.

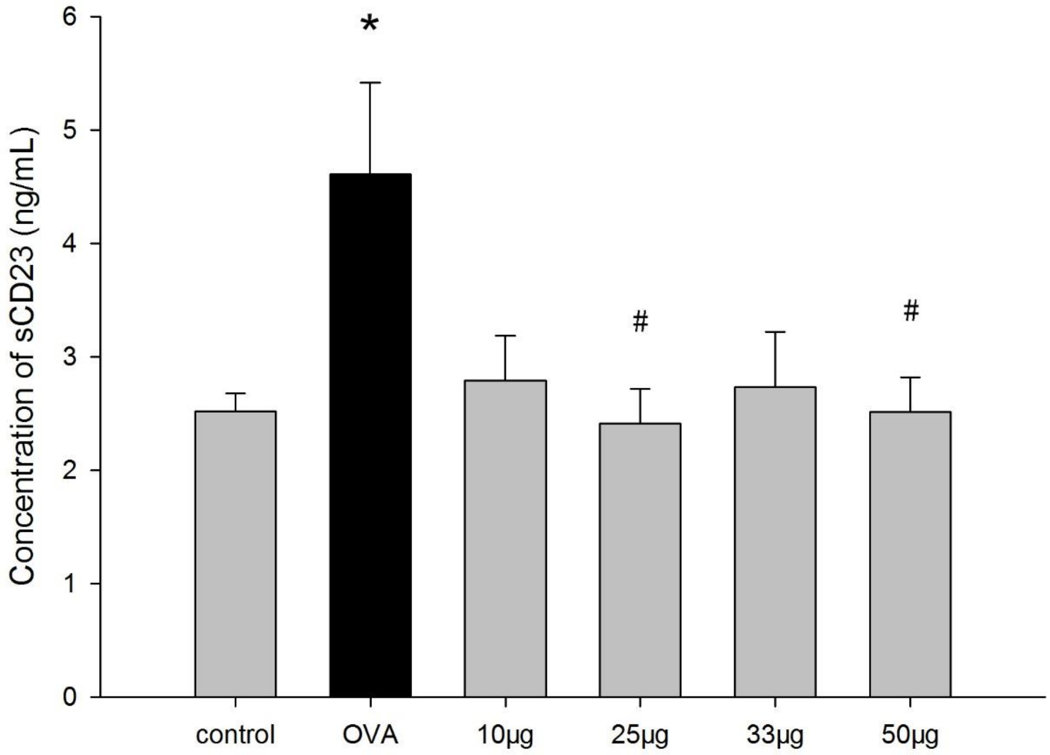

Figure S6. Concentration of soluble CD23 (sCD23) in lung homogenates from different groups of mice. The OVA-sensitized mice showed increased concentration of sCD23 when compared to the non-sensitized mice (*OVA* vs. *control*, p<0.05). However, decreased concentration of sCD23 were observed after treatment of BK-1361 at 25 or 50μg dose respectively on OVA-sensitized mice (*25, 50μg* vs. *OVA*, p<0.005), while the effects of BK-1361 at 10 and 33μg doses were not significant. Interestingly, as compared to the dosage of 25μg, no better effect was shown when the dosage of 33 or 50μg was given. Each experiment has been performed in duplicate or triplicate with n=5-6 samples per group. Result are expressed as mean ± SEM. *P<0.05 versus control. #P<0.05 versus OVA.

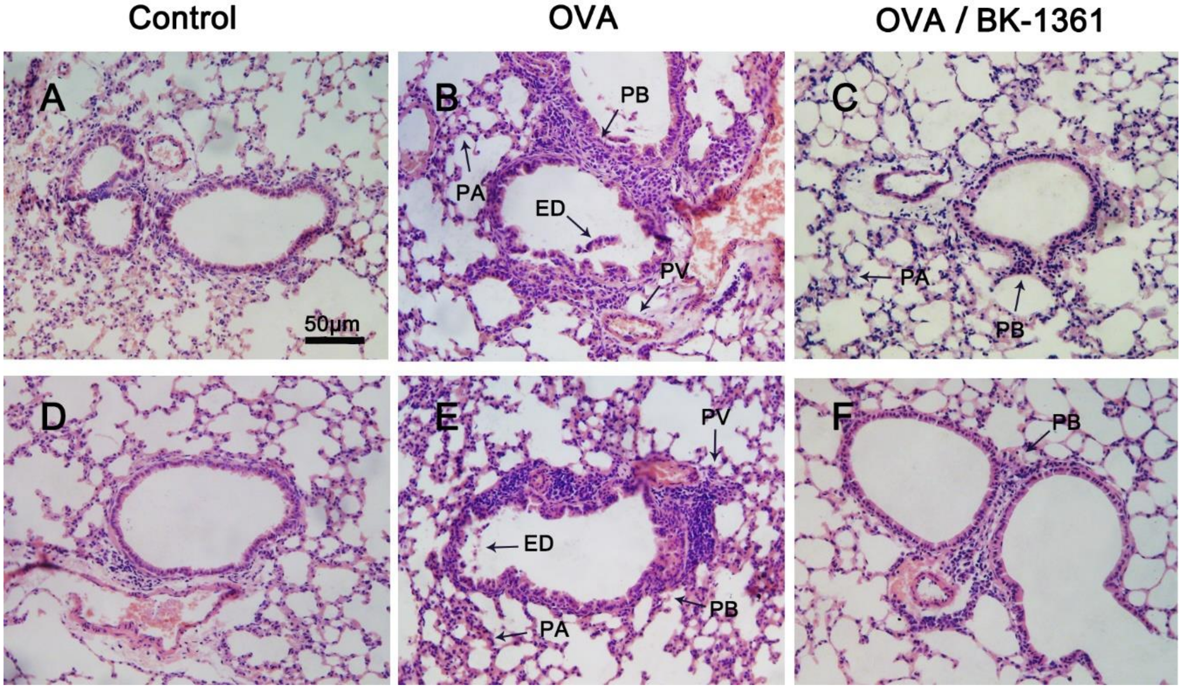

Figure S7. Representative micrograph images of lung sections stained with hematoxylin and eosin (H&E). Images A and D, B and E, C and F correspond to lung sections from control, OVA and OVA/BK-1361 (25µg/g bodyweight) groups, respectively. The bar in A (applicable to A-F) equals 50µm. The arrows in A-C point to perivascular infiltration (PV), peribronchial infiltration (PB), parenchymal infiltration (PA), epithelial damage (ED), respectively. Treatment with BK-1361 resulted in less severe PV, PA and PB, as well as an almost complete lack of ED as compared to OVA-sensitized and challenged mice alone.
